# Associations of plasma biomarkers of neurodegeneration with characteristics related to type 1 diabetes

**DOI:** 10.1002/alz.092193

**Published:** 2025-01-09

**Authors:** Nikki Delgado, Naomi Sage Chaytor, Laura Thi Germine, Zoë W Hawks, Laneé Jung, Michael Cleveland, Jane Bulger, Elizabeth Grinspoon, Kamille Janess, Martin J. Sliwinski, Ruth S Weinstock, Jasmeer P. Chhatwal, Pia Kivisäkk, Michal S Beeri, Luciana Mascarenhas Fonseca

**Affiliations:** ^1^ Elson S. Floyd College of Medicine, Washington State University, Spokane, WA USA; ^2^ Institute for Technology in Psychiatry, McLean Hospital, Belmont, MA USA; ^3^ Harvard Medical School, Boston, MA USA; ^4^ Washington State University, Pullman, WA USA; ^5^ SUNY Upstate Medical University, Syracuse, NY USA; ^6^ Jaeb Center for Health Research, Tampa, FL USA; ^7^ Pennsylvania State University, State College, PA USA; ^8^ Brigham and Women's Hospital, Boston, MA USA; ^9^ Massachusetts General Hospital, Harvard Medical School, Boston, MA USA; ^10^ Herbert and Jackeline Krieger Klein Alzheimer's Research Center, New Brunswick, NJ USA; ^11^ Old Age Research Group (PROTER), Department and Institute of Psychiatry, Faculty of Medicine, University of São Paulo, Brazil, Sao Paulo Brazil

## Abstract

**Background:**

Cognitive dysfunction is more common in individuals with type 1 diabetes (T1D) compared to the general population. Blood‐based biomarkers are accurate in identifying early signs of neurodegeneration. However, studies using these biomarkers in T1D are lacking. We examined the associations of plasma biomarkers and diabetes characteristics in adults with T1D.

**Methods:**

This study included 114 adults with T1D from the Glycemic Variability and Fluctuations in Cognitive Status in Adults With Type 1 Diabetes (GluCog) study. Plasma biomarkers were: ratio of amyloid‐β (Aβ) 42/40, neurofilament light chain (NfL), glial fibrillary acidic protein (GFAP), phosphorylated Tau181 (pTau181) using Quanterix assays, and phosphorylated Tau 217 (pTau217) using ALZpath. The coefficient of variation (%) from continuous glucose monitor readings was used to define glycemic variability (GV). Relationships between biomarkers, demographics, and diabetes characteristics were evaluated using Spearman rank correlation, Mann‐Whitney U, and Kruskal‐Wallis tests, as appropriate. Linear regression models were used to examine the associations between plasma biomarkers (dependent variable) and diabetes characteristics (that were significant in bivariate analyses) as predictors in separate models adjusted for sex, age, and education. We added the adjustment for nephropathy in models in which the biomarker was associated with this variable.

**Results:**

The sample [53% female, mean(SD) age of T1D diagnosis 20.3(12.7) years, 37% with hypertension, 11% with neuropathy, 8% with nephropathy)] had a baseline age of 49.0(14.5) years, HbA1c 7.6%(1.3), GV 36.6%(7.3). Adjusted linear regression models revealed significant associations with four biomarkers. Presence of nephropathy was associated with higher concentrations of NfL, pTau181, and pTau217 and was included in the models with these biomarkers. A lower Aβ42/40 ratio was associated with older age at diagnosis. A higher concentration of NfL was associated with higher HbA1c. A higher concentration of pTau181 was associated with higher GV. Lastly, higher pTau217 was associated with neuropathy. GFAP was not associated with any of the diabetes measures.

**Conclusion:**

These data show that plasma biomarkers of neurodegeneration are associated with key diabetes characteristics in adults with T1D, particularly HbA1c, GV, neuropathy, and nephropathy. Further research is needed to understand whether T1D characteristics mediate biomarker associations with cognitive decline.

